# The Effects of Holistic, External, and Internal Attentional Focus Instructions on Power and Kinematics of the Hang Power Snatch in Highly Skilled Weightlifters

**DOI:** 10.5114/jhk/185525

**Published:** 2024-04-15

**Authors:** Jerzy Sadowski, Paulina Szyszka, Hubert Makaruk, Marcin Starzak, Tomasz Niźnikowski

**Affiliations:** 1 Department of Sports and Training Science, Faculty of Physical Education and Health, Józef Piłsudski University of Physical Education in Warsaw, Biała Podlaska, Poland.; 2 Department of Athletics, Faculty of Physical Education and Health, Józef Piłsudski University of Physical Education in Warsaw, Biała Podlaska, Poland.; 3 Department of Gymnastics, Faculty of Physical Education and Health, Józef Piłsudski University of Physical Education in Warsaw, Biała Podlaska, Poland.

**Keywords:** training, motor performance, barbell kinematics, Olympic lifts

## Abstract

Athletes across various sports seek to enhance their power generation and force production by incorporating weightlifting exercises into their training. Therefore, integrating partial weightlifting movements could be sensible due to their simplified execution. Our research aimed to investigate which of four attentional focus strategies (external, internal, holistic, and neutral) would have the greatest impact on performance in terms of power variables for highly experienced weightlifting athletes in a practical training setting. Twelve highly skilled Olympic weightlifters volunteered for the study. They performed 48 single repetitions of the hang power snatch with each of the four attentional focus strategies. Results of the ANOVA did not reveal a significant main effect for maximum velocity, power measurement and displacement. Despite extensive research demonstrating how attentional focus affects performance differently, even among highly skilled populations, the lack of observed effects in our study underscores the challenges of conducting research in applied settings.

## Introduction

Olympic weightlifting exercises and their variations are utilized in the training programs of various sports to enhance the strength and power of athletes (Winchester et al., 2009). One of the reasons for incorporating these exercises is the importance of harmonious triple extension of the hips, knees, and ankles during the second phase of the lift in the clean, the snatch and their derivative exercises. The triple extension, as a movement pattern, has been reported to improve the transfer of training effects to the performance of jumps, sprints as well as changes of directions in real life conditions in other sports (Hori et al., 2008). Athletes representing different sports endeavor to enhance their ability to generate power and rapidly produce force by incorporating weightlifting exercises into their training (Haff and Nimphius, 2012). The snatch and the clean are highly technical, explosive, multi-joint exercises that can pose significant technical challenges. Therefore, performing partial instead of full weightlifting movements may be sensible, considering the reduced complexity of execution. It would allow athletes to refine their technique more easily and subsequently facilitate effective strength and power development. One commonly utilized variation of the snatch is the hang power snatch. The characteristic feature of this snatch is that the barbell is lifted in a short period as the athlete skips the full squat position in the snatch. Additionally, lighter loads are used in this snatch variation, allowing the barbell to achieve greater vertical speed. Considering both aspects of the snatch, it is suggested that incorporating this exercise into training induces favorable neuromuscular adaptation, enhancing power generated by the athlete's muscles (Haff and Nimphius, 2012). Power is crucial in weightlifting training and can be defined as the product of the barbell weight and its vertical velocity (barbell power) and can be used to assess exercise intensity (Bartonietz, 1996). As for the force-time curve associated with weightlifting (in the snatch or the clean), the second phase of the lift in these exercises provides the highest peak power, peak force, and the rate of force development compared to other phases (Häkkinen and Kauhanen, 1986; Souza et al., 2002).

One factor that has received considerable support for its influence on peak power, force, and the rate of force development is attentional focus. Studies have shown that instruction promoting different types of attentional focus can have a significant impact on motor performance and learning (Wulf, 2013). Furthermore, evidence suggests that instructions given during exercises affect how athletes develop concentration-oriented strategies during competitions (Porter et al., 2010). The research has primarily compared the effects of instructions that promote internal or external attentional focus.

The research on attentional focus has shown compelling evidence that instructing individuals to focus externally (i.e., focusing on the movement outcome) leads to more efficient and effective performance and learning effects compared to instructing them to focus on their body movements (i.e., promoting internal focus). The benefits of external focus instructions have been demonstrated in a wide range of motor tasks such as golf, tennis, baseball, volleyball, soccer, and basketball (Wulf, 2013). To account for these findings, Wulf et al. (2001) proposed the “constrained action hypothesis. Wulf and Prinz (2001) indicate that external attentional focus induces more automatic control of movement, which results in enhanced motor performance. In contrast, directing attention internally has been shown to result in a conscious type of control that restricts, or constrains, the automatic control processes of a movement (Wulf et al., 2001). Research has largely demonstrated that focusing outside the body and on the intended outcome of the movement (i.e., external focus) yields better results in learning and movement performance than focusing on body parts, specific movements, or mechanics (i.e., internal focus) (Neumann, 2019). External attentional focus has been shown to result in increased movement efficiency (e.g., reduced muscle activity, higher peak force), movement performance (e.g., better balance, greater accuracy, greater speed, better endurance), a better movement form, as well as more automatic and fluent movements (Artymiak et al., 2017; Razaghi et al., 2020; Wulf and Lewthwaite, 2016).

The above-mentioned studies have shown the superiority of external focus over internal focus among novice athletes and experts, while Chua et al. (2021) suggested that the benefit of external focus was independent of the skill level. However, it is worth noting that others have claimed the opposite when examining immediate motor performance (Nicklas et al., 2022). Additionally, Porter and Smis (2013) suggested that in highly skilled athletes, a change in attentional focus might lead to a decrease in performance. They noted that any change in attentional focus resulted in a deterioration of sprint results over short distances. The best results were achieved when athletes did not focus their attention at all. Similar suggestions were presented by Wulf (2008), who, while studying acrobats, noted that a lack of attentional focus led to increased movement adjustments, indicating increased movement automatism. Therefore, some scientists have questioned the effectiveness of using external and internal attentional focus and proposed a holistic approach to extend beyond the internal/external paradigm, which, according to the definition of Becker et al. (2019), directs attention to the overall feeling of movement or sensations associated with it. This focus differs from internal focus as it does not direct attention to a specific body movement (e.g., leg extension). One could argue that this type of attentional focus is an extension of external focus, as the emphasis is placed on the intended outcome of the movement. Interestingly, holistic attentional focus is often dominant in the instructions given to athletes by their coaches, as well as among high-performance athletes (Zhuravleva et al., 2022; Zhuravleva and Aiken, 2023). Becker et al. (2019) studied the impact of holistic, internal and external attentional focus during the standing long jump. It was shown that holistic attentional focus and external attentional focus led to greater performance than the internal attentional focus condition. Holistic and external focus did not significantly differ from each other. Those authors argued that instead of external attentional focus, holistic attentional focus could be applied. It was observed that focusing on the overall feeling of movement (e.g., smoothness, fluency, strength, etc.) led to significantly better results in tasks related to long jumps, free throws in basketball, and putting in golf (Mullen and Hardy, 2010). Moreover, it was found that the goals of the holistic process allowed for more automatic processing, while focusing on partial processes disrupted these movements (Masters, 1992). This is consistent with previous research that showed the benefits of a holistic approach (Abedanzadeh et al., 2022; Becker et al., 2019). Holistic attentional manipulation also seems consistent with the limited action hypothesis and external focus. However, research also suggests that experienced athletes use attentional focus differently than novices (Kostrzewa et al., 2020; Porter and Sims, 2013; Raisbeck et al., 2020; Yamada et al., 2020). Expert performers use multiple attentional focus strategies (Fairbrother et al., 2016) or prefer not to change attentional focus (Porter and Sims, 2013).

One of the primary responsibilities of weightlifting coaches is to create a practice environment that enhances performance. One challenge that coaches encounter is how to create the best instructions for practice of the snatch that will facilitate power development. In weightlifting, studies on attentional focus suggest that coaches should develop outcome-directed instructions rather than focusing on movement mechanics (Camargo, 2014; Everett, 2009). This enables the athlete to free up attentional resources through the application of fast, automatic control processes. Verbal coaching guidance in Olympic weightlifting is based on coaches' experience and observations (Takano, 1993; Tysz, 2010). For instance, Everett (2009) suggests instructing the athlete on what to do rather than allowing the barbell to deviate from the body: “We can instruct the athlete to maintain a more upright posture, actively pulling the bar towards the torso with the lats or direct the elbows upward and outward”. Furthermore, Takano (1993) states that “The critical point to direct the athlete's attention during the extension of the arms is to focus on lifting the elbows high and wide, rather than lifting the barbell. In all movements, emphasis should be placed on the movement of body parts, not the movement of the barbell”. Improving instructional models in coaching education can impact sports performance, increase the effectiveness of exercises performed, and reduce the risk of injuries. Several studies provide information on the benefits of using the appropriate attentional focus strategy, with researchers mainly investigating the impact of external and internal attentional focus strategies (Amri-Dardari et al., 2022; Furuhashi et al., 2023). In contrast to the growing body of evidence showing that external and internal attentional focus strategies have distinct advantages, some new strategies for guiding athletes have appeared. To date, only few studies have investigated the effects of a holistic strategy of attentional focus among highly skilled athletes, particularly within the applied context (Zhuravleva and Aiken, 2023; Zhuravleva et al., 2023). Therefore, our research aimed to examine which of the proposed four attentional focus strategies (external, internal, holistic, and neutral) would yield the greatest performance effects in terms of power variables for highly experienced weightlifting athletes within an applied weightlifting context.

## Methods

### Participants

Twelve highly skilled Olympic weightlifters (n = 6 women: body height = 172.67 ± 3.2 cm; body mass = 69.83 ± 6.55 kg; n = 6 men: body height = 180.33 ± 8.31 cm; body mass = 96.83 ± 18.51 kg) volunteered to participate in the study (mean age = 22.75 ± 2.0 years). The athletes' training experience varied from four to nine years (mean training experience = 6.75 ± 2.6 years). These individuals were medalists in national championships and represented their country at the international level.

Participants did not report upper extremity injuries over the previous three months. All participants were involved in the study voluntarily, and they delivered written informed consent prior to the commencement of the experiment. The study was conducted following the principles of the Declaration of Helsinki. The Scientific Research Ethics Committee of the Józef Piłsudski University of Physical Education in Warsaw provided ethical approval (approval code: SKE 01-42/2023; approval date: 16 June 2023).

The sample size for the present study was based on sample sizes and analyses of similar studies (North et al., 2019, n = 10; Porter et al., 2020, n = 12). Power analysis of the study using G*Power Version 3.1.9.4 (Faul et al., 2007) showed that with an estimated moderate effect size, a minimum of twelve participants were required (effect size = 0.25, power = 0.80, *p* = 0.05). Therefore, the recruited sample of 12 participants was considered appropriate for this investigation.

### Experimental Approach to the Problem

This study utilized a within-participant design to observe performance differences in the hang power snatch between different attentional focus instructions. Participants performed 4 sets of 3 repetitions with external, internal, holistic, and neutral attentional focus instructions. All participants performed a block of warm-up repetitions, followed by 4 sets of 3 repetitions per attentional focus conditions. The attentional focus conditions were counterbalanced to avoid potential order effects. Additionally, the experiment occurred on four separate training days (i.e., Day 1, Day 7, Day 14, Day 21) over a month to avoid any possible carryover effects from the attentional focus instructions. A different attentional focus condition was applied during each training day. Barbell kinematics were recorded using the WL Analysis application.

During the pilot study stage, the results obtained using the WL Analysis application were compared with those obtained from the Vicon system. The analysis was conducted on 144 pairs of maximum speed values obtained in the same snatch approaches. No statistically significant differences between the results were found (U Mann-Whitney test *p* = 0.206 and Kolmogorov-Smirnov test *p* > 0.10). Additionally, to assess the reliability of measurements using the application, an analysis was conducted using the Interclass Correlation Coefficient, yielding a value of 0.84.

### Procedures

The study participants performed a standardized warm-up. The experimental task consisted of 48 single repetitions of the hang power snatch at 80% of the participant’s self-reported 1 repetition maximum (1-RM). Four-minute rest intervals were provided between each of the repetitions. During the external condition, the researcher instructed the participant to “focus on moving the barbell back and up rapidly”. For the internal condition, the participant was instructed to “focus on moving his/her elbows high and to the side rapidly”. During the holistic condition, participants were instructed to “focus on feeling as explosive as possible”. For the neutral condition, participants were instructed to “focus on performing the snatch to the best of their ability”. The choice of instruction given to athletes was consistent with the most common instructions that weightlifting coaches use.

### Statistical Analyses

The four trials completed in each of the four conditions across each week were used for each participant, resulting in 48 trials per athlete. The Statistical Package for the Social Sciences (SPSS, version 29.0.0; IBM, Armonk, NY, USA) was used for the statistical analysis. Analysis of variance (ANOVA) with repeated measures was used to assess the differences among the four experimental conditions for maximum velocity, power, and displacement. Prior to analysis, the Mauchly’s test for sphericity was performed and Greenhouse-Geisser correction was applied when sphericity was violated. The effect size was calculated using partial eta squared. The criterion for significance was set using an alpha level of *p* ≤ 0.05.

## Results

Results of ANOVA did not reveal a significant main effect for condition for maximum velocity (F(3, 33) = 1.215, *p* = 0.320, ηp2 = 0.099). No significant differences were found among conditions. Additionally, the results of ANOVA for power measurements did not indicate a significant main effect for condition (F(3, 33) = 2.096, *p* = 0.120, ηp2 = 0.160). Finally, ANOVA for displacement did not reveal a significant main effect (F(3, 33) = 0.835, *p* = 0.485, ηp2 = 0.071). Additionally, 4 (condition) x 4 (day) ANOVA was performed for each dependent variable to determine whether there was a possible order (day) effect. No statistical differences were observed and did not alter the results of the previous analyses.

**Figure 1:**
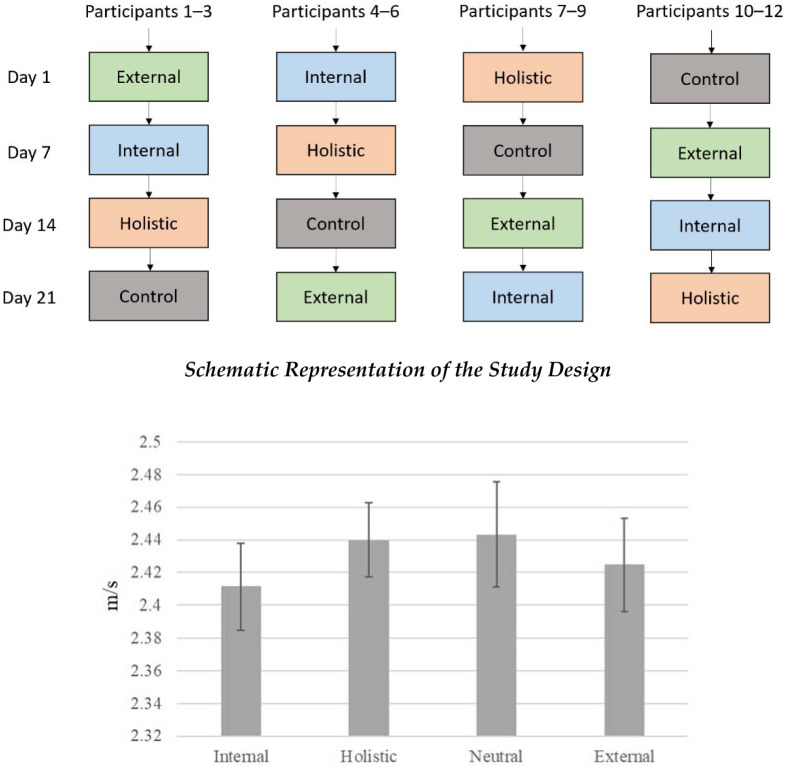
Comparison of maximum barbell velocity among the studied conditions. *Note: Error bars represent standard error (SE)*.

**Figure 2:**
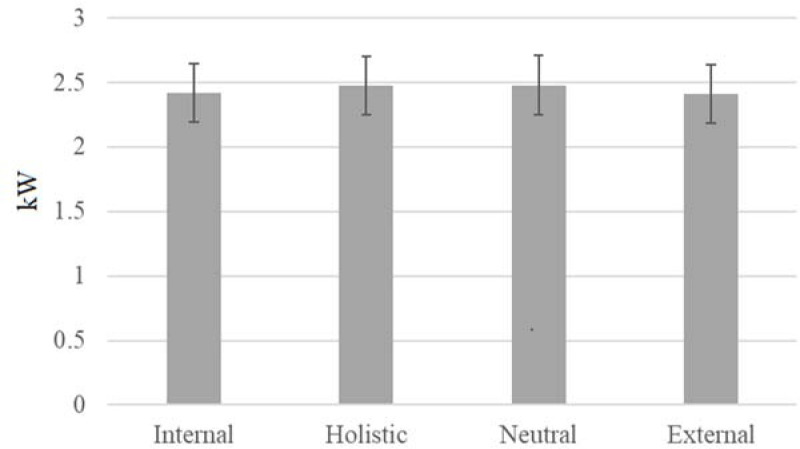
Comparison of power among the studied conditions. *Note: Error bars represent standard error (SE)*.

**Figure 3:**
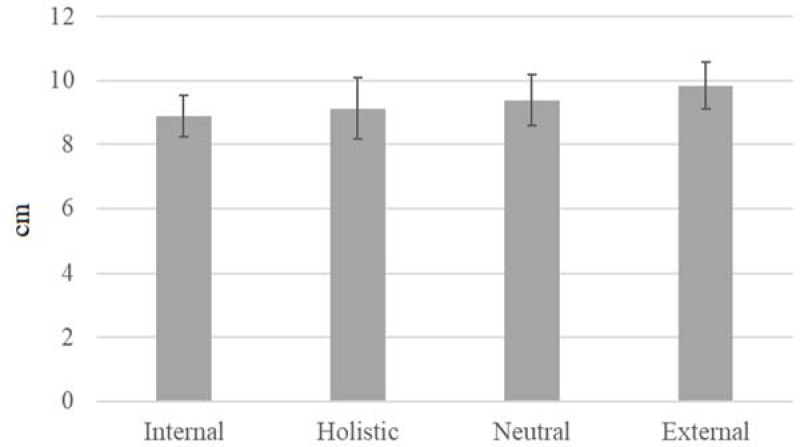
Comparison of horizontal displacement of the barbell among the studied conditions. *Note: Error bars represent standard error (SE)*.

## Discussion

Creating a practice environment that best facilitates the performance of athletes is a primary aspect for weightlifting coaches. Given the previous evidence highlighting the influence that instructions have on performance, the purpose of the present study was to investigate different instructional strategies for highly skilled weightlifting athletes. Specifically, the goal was to investigate how instructions that directed attention externally, internally, holistically, and neutrally influenced the performance of the power snatch as measured by the barbell kinematics. Much of the Olympic weightlifting coaching literature suggests providing instructions that would likely lead to adopting internal attentional focus (Everett, 2009; Takano, 1993; Tysz, 2010). However, a significant body of scientific evidence has demonstrated that instructions promoting external attentional focus results in superior motor performance (Chua et al., 2021). Furthermore, recent research has also suggested that instructions promoting holistic attentional focus can have beneficial performance effects (Becker et al., 2019). Therefore, it was hypothesized that instructions promoting internal focus would lead to significantly worse performance relative to neutral, external, and holistic attentional focus promoting instructions. However, the results of the current study did not support this hypothesis and did not reveal any statistically significant differences in performance among any of the different instructional strategies. Considering the above, we cannot provide clear recommendations on which instruction strategies weightlifting coaches should use during power training.

The most prominent explanation for the observed motor performance benefits is the constrained action hypothesis (Wulf et al., 2001). Based on this hypothesis, focusing externally allows the motor system to naturally self-organize, whereas focusing internally constrains the motor system and results in the detrimental conscious control of movements. Initial support for this explanation comes from research demonstrating that focusing internally during a balancing task led to lower response frequencies relative to external focus (Wulf et al., 2001). Additional support for the constrained action hypothesis showed that internal focus led to greater co-contraction between the agonist and antagonist muscles, resulting in inefficient motor control (Lohse et al., 2011). In contrast, external focus led to lower muscular activity, greater force production (Lohse et al., 2011) and more efficient neural activity (Kuhn et al., 2017).

Since then, a significant body of research has demonstrated the superiority of external attentional focus over internal focus (Chua et al., 2021). A great portion of this research has investigated how attentional differences influence motor performance and learning within novice populations. However, the external attentional focus effect has been demonstrated within highly skilled populations as well (Chua et al., 2021; Hosseiny et al., 2014; Makaruk et al., 2013; Porter et al., 2013) and has been reported to generalize across different skill levels. Despite finding support for external attentional focus in highly skilled populations, some research has not found that external focus led to superior performance among experts (Wulf, 2008). These findings are more closely in line with the results of the present study. Although in the present study barbell velocity was trending towards a lower maximum velocity under the internal condition compared to the other conditions, non-significant differences were observed. Previous researchers have argued the traditional internal/external examination of the attentional focus effect may be limited by this dichotomous investigation (Becker et al., 2019; Woodard et al., 2021; Zhuravleva et al., 2022; Zhuravleva and Aiken, 2023). That is, there are more ways to direct attention beyond just internal and external focus. For example, Woodard et al. (2021) demonstrated that highly skilled performers utilize multiple attentional strategies to accomplish their movement goal. Furthermore, Zhuravleva et al. (2022) and Zhuravleva and Aiken (2023) argued a similar notion demonstrating that skilled athletes utilized holistic attentional focus more frequently than traditional internal or external focus. However, while several studies have indicated that external focus may not enhance performance in highly skilled populations, it has been observed that internal attentional focus hinders performance, relative to neutral focus (Wulf, 2008). Thus, while the current experiment somewhat aligns with investigations in highly skilled populations observing a lack of performance benefits when utilizing external focus, the present study is not congruent with most studies showing the detrimental effects of internal focus.

It is possible that the lack of observed differences in the present study could be due to methodological limitations. First, the WL Analysis application was used to assess the hang power snatch. This is commonly utilized software within Olympic weightlifting training environments. While this software has been internally validated in relation to the three-dimensional motion capture system, it has received minimal scientific attention assessing validity and reliability. Thus, perhaps the performance metrics assessed in the current study were not sensitive enough to determine statistical differences. Additionally, the relatively small sample size used in the current study is another limitation that could explain the lack of observed differences. Both of these limitations can increase the probability of a false negative type II error (Vadillo et al., 2016). Thus, the results of the present study should be interpreted cautiously. In other words, while the different types of instructions may not seemingly have an effect on power snatch performance of highly skilled weightlifters, perhaps low statistical power is the reason for the non-observed statistical differences.

Overall, this study investigated the effects of instructions promoting internal, external, holistic, and neutral attentional focus on power snatch performance of highly skilled weightlifters within an applied context. No significant differences were observed for any of the performance metrics among any of the attentional focus conditions. Given the depth of research demonstrating how attentional focus differentially affects performance, even within highly skilled populations, the lack of observed effects within the present study highlights the challenge of conducting research within applied settings. Further research on a larger sample should be carried out in order to compare the effects of holistic attentional focus in athletes with different skill levels (novices, sub-elite and elite athletes). Additionally, the validity and reliability of WL Analysis software for performance analysis should be determined as it may provide a simple and affordable way of collecting performance data for Olympic weightlifting training purposes.
